# Three Growth Factors Induce Proliferation and Differentiation of Neural Precursor Cells *In Vitro* and Support Cell-Transplantation after Spinal Cord Injury *In Vivo*

**DOI:** 10.1155/2020/5674921

**Published:** 2020-06-17

**Authors:** Alexander Younsi, Guoli Zheng, Moritz Scherer, Lennart Riemann, Hao Zhang, Mohamed Tail, Maryam Hatami, Thomas Skutella, Andreas Unterberg, Klaus Zweckberger

**Affiliations:** ^1^Department of Neurosurgery, University of Heidelberg, Im Neuenheimer Feld 400, 69120 Heidelberg, Germany; ^2^Department of Neuroanatomy, Institute for Anatomy and Cell Biology, University of Heidelberg, Im Neuenheimer Feld 307, 69120 Heidelberg, Germany

## Abstract

Stem cell therapy with neural precursor cells (NPCs) has the potential to improve neuroregeneration after spinal cord injury (SCI). Unfortunately, survival and differentiation of transplanted NPCs in the injured spinal cord remains low. Growth factors have been successfully used to improve NPC transplantation in animal models, but their extensive application is associated with a relevant financial burden and might hinder translation of findings into the clinical practice. In our current study, we assessed the potential of a reduced number of growth factors in different combinations and concentrations to increase proliferation and differentiation of NPCs in *vitro*. After identifying a “cocktail” (EGF, bFGF, and PDGF-AA) that directed cell fate towards the oligodendroglial and neuronal lineage while reducing astrocytic differentiation, we translated our findings into an *in vivo* model of cervical clip contusion/compression SCI at the C6 level in immunosuppressed Wistar rats, combining NPC transplantation and intrathecal administration of the growth factors 10 days after injury. Eight weeks after SCI, we could observe surviving NPCs in the injured animals that had mostly differentiated into oligodendrocytes and oligodendrocytic precursors. Moreover, “Stride length” and “Average Speed” in the CatWalk gait analysis were significantly improved 8 weeks after SCI, representing beneficial effects on the functional recovery with NPC transplantation and the administration of the three growth factors. Nevertheless, no effects on the BBB scores could be observed over the course of the experiment and regeneration of descending tracts as well as posttraumatic myelination remained unchanged. However, reactive astrogliosis, as well as posttraumatic inflammation and apoptosis was significantly reduced after NPC transplantation and GF administration. Our data suggest that NPC transplantation is feasible with the use of only EGF, bFGF, and PDGF-AA as supporting growth factors.

## 1. Introduction

In recent years, stem cell therapy has been introduced as a promising treatment strategy to improve neuroregeneration and functional recovery after spinal cord injury (SCI) [1, 2]. SCI remains a disastrous event with limited spontaneous recovery, often disabling affected patients for life and representing a severe burden to the individual fates as well as health care systems [3–5]. Especially neural stem- or precursor cells (NPCs) are considered promising candidates for application in such stem cell treatments with the potential to differentiate into neurons or oligodendrocytes and thus to regenerate the damaged neural tissue [6–8]. Furthermore, it has been reported that NPCs release neurotrophic factors [9] and modify the immune environment [10]. However, the survival of transplanted NPCs is generally low, and it remains especially challenging to induce their differentiation towards the neuronal or oligodendroglial lineage [11]. As a result, efforts have been made to improve engraftment and differentiation of NPCs with growth factors, and different combinations and concentrations of such proteins or steroid hormones have been assessed. Hereby, a larger number of different growth factors and a higher concentration typically resulted in improved proliferation and survival of NPCs [12, 13], creating a severe financial burden for researchers. For this reason, transplantation strategies incorporating the use of many growth factors might be impractical considering possible translation into clinical practice [14].

The goal of our study, therefore, was to identify a cost-effective combination and concentration of growth factors, suitable to improve NPC survival and differentiation *in vitro* and translate our findings into an animal model of SCI *in vivo*. While a wide range of growth factors with potentially beneficial properties is available, we aimed to select proteins that would specifically enhance NPC proliferation, fostering neuroprotective effects [15] as well as differentiation towards the neuronal and oligodendroglial lineage for, e.g., enhanced remyelination [16] and neuronal relay formation [12]. Because of reports that have linked NPC-derived astrocytes to increased astrogliosis and exacerbation of functional outcome [17], we additionally sought to determine a growth factor combination that in particular would not promote astrocytic differentiation.

In this context, the peptides epidermal growth factor (EGF) and basic fibroblast growth factor (bFGF) are of special interest. Being potent mitogenic factors, they both have shown to expand the subventricular zone cell population *in vitro* [18], with bFGF more specifically increasing the proliferation of NPCs [19] and leading to reduced adult neuronal cell death *in vivo* [20]. Considering our study requirements, we selected EGF and bFGF as the minimal growth factor combination for NPC proliferation and differentiation. While a concentration of 20 ng/ml is mostly used for these proteins in the literature [21–23], few reports exist on the use of a lower EGF/bFGF concentration (10 ng/ml) as well [24–26]. We therefore sought to assess the normal as well as the lower EGF/bFGF concentration in our experiment. Due to its role in promoting the proliferation of bipotential progenitors [27] and increasing the survival of differentiated oligodendrocytes [28], we chose to further expand our growth factor combination by the platelet-derived growth factor ligand AA (PDGF-AA). This, in particular, is because PDGF-AA which is secreted by type-1 astrocytes has synergistic effects with bFGF on the proliferative response of adult oligodendrocyte progenitors [29].

We hypothesized that a growth factor cocktail consisting of either EGF and bFGF alone or in combination with PDGF-AA would have sufficient properties to enhance proliferation of NPCs as well as their differentiation into neurons and oligodendrocytes *in vitro*. In addition, we sought to translate our findings into an animal model of cervical clip contusion/compression SCI, verifying survival and differentiation of transplanted NPCs *in vivo*.

## 2. Materials and Methods

### 2.1. Isolation and Cultivation of NPCs

**Figure 1:**
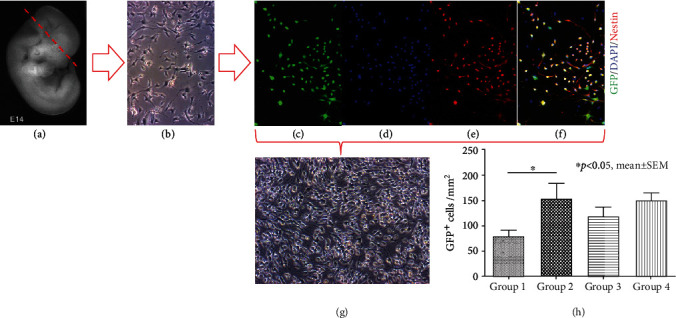
Generation, cultivation, characterization and proliferation of NPCs *in vitro*. (a) Embryo of a GFP-expressing Wistar rat before schematic dissection of the cortical hemispheres [56]. (b) Phase contrast microscopy of a 2-day old NPC monolayer culture. (c-f) Colocalization of GFP^+^ NPCs (green) with markers for intact nuclei (DAPI, blue) and stem cells (Nestin, red). (g) Phase contrast microscopy of an NPC monolayer culture after 7 days of cultivation. (h) GFP^+^ NPCs quantified after 7 days of incubation with the different growth factor concentrations/combinations in groups 1-4.

Two-week old embryos (E14) of green fluorescent protein (GFP) expressing transgenic Wistar rats (The Jackson Laboratory, USA) were used for primary NPC isolation. Embryos were washed with cold phosphate-buffered saline (PBS^−^, w/o Ca^2+^, and Mg^2*+*^; Gibco, USA), their cortical hemispheres were carefully dissected, and the meninges were removed (Figures 1(a) and 1(b)). The cleaned cortices were manually cut to small pieces and washed with warm PBS^−^, then incubated with 0.05% trypsin/ethylenediaminetetraacetic acid (EDTA) and 0.2% desoxyribonuclease (DNase) I (both Thermo Fisher Scientific, USA) for 6 min at 37°C in a water bath. The enzymatic reaction was neutralized by the addition of Dulbecco's Modified Eagle's Medium (DMEM/F12, Thermo Fisher Scientific, USA) with 10% fetal bovine serum (FBS, Thermo Fisher Scientific, USA), and the tissue pieces were manually dissociated to a single cell suspension by pipetting up and down 10 times before centrifugation (6 min at 1,200 rpm). For expansion, NPCs were plated on tissue culture plates that were precoated with 20 *μ*g/ml poly-l-ornithine (Sigma-Aldrich, USA) and 5 *μ*g/ml laminin (Sigma-Aldrich, USA) at a density of 1.5 × 10^4^ cells/cm^2^ in 1.5 ml growth medium containing DMEM/F12 with sodium bicarbonate and L-glutamine (Gibco, USA), 1% penicillin/streptomycin, 1x N2 supplement (both Gibco, USA), 20 ng/ml bFGF, and 20 ng/ml EGF (both Sigma-Aldrich, USA) (Figure 1(c)). They were incubated in a humidified incubator at 37°C with 5% CO_2_ and split when a confluence of 80–90% was reached. Viability of the NPCs as well as their stem cell characteristics was successfully assessed before further use (Figures 1(d)–1(g)).

### 2.2. Study Design and Experimental Groups *In Vitro*

Based on the minimal requirements for NPC proliferation and differentiation, we created four distinct experimental groups with different growth factor concentrations/combinations for our *in vitro* experiment (Table 1): no growth factors (group 1; control group), 10 ng/ml EGF + 10 ng/ml bFGF (group 2; minimum concentration/normal combination group), 20 ng/ml EGF + 20 ng/ml bFGF (group 3; normal concentration/combination group), and 20 ng/ml EGF + 20 ng/ml bFGF + 6 ng/ml PDGF-AA (Sigma-Aldrich, USA; group 4; normal concentration/enhanced combination group). NPCs at the third passage (p3) with a density of 2 − 3 × 10^5^ cells/ml were incubated with the corresponding growth factor combination/concentration groups in DMEM/F12 with sodium bicarbonate and L-glutamine, 1% penicillin/streptomycin and 1x N2 supplement in a humidified incubator at 37°C with 5% CO_2_ for seven days. Medium change (50%) was performed daily. After 7 days, the cells were fixed with 4% paraformaldehyde (PFA) (Santa Cruz, USA) for 20 minutes and washed with PBS before they were subjected to immunofluorescence staining. All experiments were performed in triplicate on 24-well plates.

### 2.3. Animals, Study Design, and Experimental Groups *In Vivo*

The most promising growth factor concentration/combination from the *in vitro* part of our study was translated into an *in vivo* animal model of cervical SCI to test whether it would be sufficient for NPC survival and differentiation. To this end, a total of 24 female Wistar rats (250 g; Charles River Laboratories, Germany) were randomly assigned to three treatment groups: Group 1 (NPCs + growth factors; *n* = 8), group 2 (vehicle; *n* = 8), and group 3 (sham; *n* = 8). A treatment group with NPCs only (without growth factors), not relevant for the primary aim of the *in vivo* part of our study, was omitted due to ethical considerations. NPCs were transplanted 10 days after injury, and intrathecal administration of growth factors was simultaneously initiated. As a standard for immunosuppression, minocycline (50 mg/kg s.c.; Ratiopharm, Germany) was administered daily from 2 days prior to 10 days after transplantation and daily injections of cyclosporin (10 mg/kg s.c.; Sigma-Aldrich, USA) were initiated on the day of transplantation until the end of the experiment in all groups. Functional outcome was assessed weekly, and the experiment was terminated with perfusion of all animals 8 weeks after SCI. All surgical and neurobehavioral procedures were blinded, and all experimental protocols were approved by the Animal Care Committee of the federal government.

### 2.4. Compression/Contusion Injury, Transplantation of NPCs, and Growth Factor Application

We used a contusion/compression model with a 28-g modified aneurysm clip (Fehlings Laboratory, Canada) to induce SCI at the C6 level as previously described [30]. For all surgical procedures, animals were anesthetized with 1.5% isoflurane and a 1 : 1 mixture of O_2_ and N_2_O. Postoperatively, animals were subjected to intensive care and received buprenorphine (0.05 mg/kg s.c.; Bayer, Germany) as well as meloxicam (2 mg/kg s.c.; Boehringer-Ingelheim, Germany) for 3–5 days. Fluids and nutritional support were administered to all injured animals. An antibiotic prophylaxis (moxifloxacin, 4 mg/kg p.o.; Alcon, USA) was given for 7 days, and bladders were manually squeezed three times per day until bladder reflexive function had recovered. Animals were housed in a 12 h light-dark cycle at 26°C with food and water ad libitum.

For transplantation of NPCs, animals were anesthetized as described above, and the dura at the C6 level was microsurgically reexposed. 4 × 10^5^ NPCs diluted in 8 *μ*l growth medium were injected into the spinal cord at four sites (i.e., 2 *μ*l per site), bilaterally 2 mm rostral and caudal to the epicenter of the lesion at a depth of 1.5 mm, using a stereotactic injector with a Hamilton syringe (Hamilton Company, Switzerland) and 35 g microneedle (World Precision Instruments, Germany) at a rate of 5 nl/s. Animals in group 2 (vehicle) received the same amount of growth medium without NPCs. During the same surgery, a T1 laminectomy was performed and a rat intrathecal microcatheter (Alzet, USA), connected to a subcutaneous osmotic micropump (Alzet, USA) was subdurally placed with its open end over the epicenter of the lesion. This pump was used for continuous intrathecal administration of the most promising growth factor combination tested in the *in vitro* part of our study, with a concentration adapted to the *in vivo* use (30 *μ*g/ml EGF, 30 *μ*g/ml bFGF, and 1 *μ*g/ml PDGF-AA) in all injured animals for 7 days.

### 2.5. Retrograde Fiber Labeling and Functional Outcome

We performed retrograde fiber labeling with Fluorogold (FG; fluorochrome, USA) 7 days prior to the end of the experiment in 5 randomly selected animals per group. To this end, the T2 lamina was removed, the dura was exposed, and two injections of 0.5 *μ*l FG (4%, dissolved in PBS) were given 0.5 mm bilateral to the posteromedian vein at a depth of 1.2 mm using the stereotactic setting mentioned above.

In order to assess functional recovery of the hindlimbs, the Basso-Beattie-Bresnahan locomotor rating scale (BBB) was performed weekly. To this end, rats were placed into an open field for 4 min, and hindlimb joint movement, coordination, and weight support were evaluated by 3 independent observers using a rating scale from 0 to 21 points [31]. In addition, the CatWalk automated quantitative gait analysis system (Noldus Ltd., Netherland) was used every other week to measure parameters of the movement pattern with a focus on dynamic and front limb associated values (average speed, stride length of the front limbs). Animals had to perform a minimum of 3 quantifiable runs for each measurement, and the data were processed and analyzed by the CatWalk software. Baselines for the BBB and the CatWalk were acquired for all animals prior to SCI.

### 2.6. Animal Perfusion, Tissue Processing, and Immunofluorescence Staining

For perfusion and tissue processing 8 weeks after SCI, animals were deeply anesthetized with isoflurane (4%) and transcardially perfused with 50 ml 0.1 M cold PBS followed by 150 ml PFA (4% in 0.1 M PBS). Spinal cords and brains were removed and fixed in 4% PFA for 24 h before cryoprotection in 30% sucrose for 48 h. Spinal cord segments of 2 cm length centered around the lesion epicenter were dissected and embedded into tissue embedding medium (Sakura Finetek Europa B.V., Netherlands) on dry ice. Consecutive cross-sections of the spinal cord pieces (every 240 *μ*m) and the brains (continuously) with a thickness of 30 *μ*m were cut with a cryostat (Leica Biosystems, Germany), dried and stored at −80°C until further use.

For immunofluorescence staining, the cells (*in vitro*) or tissue sections (*in vivo*) were first blocked with 1% bovine serum albumin (BSA), 5% milk powder, and 0.3% Triton-X100 (all Sigma-Aldrich, USA) in 0.1 M PBS at room temperature for 1 h and then incubated with the following primary antibodies diluted in the same blocking solution at 4°C overnight: anti-DCX (mouse, 1 : 400; Santa Cruz Biotechnology, USA), anti-NeuN (rabbit, 1 : 500; Merck-Millipore, Germany), anti-APC (mouse, 1 : 200; Merck-Millipore, Germany), anti-NG2 (rabbit, 1 : 400; Merck-Millipore, Germany), anti-GFAP (mouse, 1 : 1000; Merck-Millipore, Germany), anti-Nestin (mouse, 1 : 200; Merck-Millipore, Germany), anti-MBP (mouse, 1 : 100; Santa Cruz Biotechnology, USA), anti-Iba1 (rabbit, 1 : 1000; Thermo Fisher Scientific, USA), and anti-Caspase-3 (rabbit, 1 : 200; Cell Signaling, USA). Isotype controls with nonspecific immunoglobulin at the same concentration were performed to ensure specificity of the antibody staining (data not shown).

After removal of the primary antibody dilution, the secondary antibodies (Alexa Fluor 568 goat anti-mouse, 1 : 500; Alexa Fluor 647 goat anti-rabbit, 1 : 500; both Thermo Fisher Scientific, USA), diluted in blocking solution without Triton-X-100, were applied for 1 h at room temperature. DAPI (1 : 10000; Sigma-Aldrich, USA) was added for 30 min before the cells/tissue sections were subjected to imaging analysis.

### 2.7. Imaging Analysis

All images were obtained using a confocal laser scanning microscope (LSM 700, Carl-Zeiss, Germany). Images of each well (*in vitro*) or tissue section (*in vivo*) were taken at 10x magnification in the 8-bit format with the tile scan function (speed of 6, gain of 800). Four wavelength channels (DAPI 405 nm, GFP 488 nm, Alexa Fluor 568 nm, and Alexa Fluor 647 nm) with light transmission ranging from 2.8% to 100% were used. For quantitative assessment of the proliferation and differentiation of NPCs *in vitro* and *in vivo* (GFP, DCX/NeuN, NG2/APC, Nestin), apoptosis (Caspase-3) as well as descending tract regeneration (FG) semi-automatic cell counting was performed by three independent and blinded investigators. To this end, we used an algorithm for ImageJ2 (National Institute of Health, Bethesda, USA) as previously described [10]. Briefly, the images were split into single channels, a Gaussian-filter (Sigma: 10.00) was applied to reduce background noise and the Isodata thresholding algorithm was used to transform selected regions of interest (ROIs) into binary images.

For the analysis of NPCs *in vitro*, ROIs were placed around the whole well. For the analysis of NPCs *in vivo* as well as apoptosis, ROIs consisted of the entire spinal cord without the cyst and the autofluorescence border. For the analysis of descending tract regeneration, ROIs were placed around the entire brainstem.

Positive-labelled cells with signals above specific thresholds were then counted in the selected ROI with the “Analyze Particles” function. Next, binary images were recombined using the “Image Calculator” function, and the costained cells within the same ROI were counted. To avoid inclusion of artefacts, only structures with an area of 50-2000 *μ*m^2^ were considered.

For the *in vitro* analysis of NPCs, results are given either as cells/mm^2^ (proliferation) or in proportion (fold) to the control group (differentiation). For the *in vivo* analysis of NPCs, the total number of cells per animal was further estimated by multiplying the total cell count of all consecutive tissue sections with the intersection distance (240 *μ*m) divided by the section thickness (30 *μ*m) and results are given either as a percentage of the initial cell transplant (survival) or as the total number of cells (differentiation) per group. For the analysis of apoptosis *in vivo*, the total number of Caspase-3^+^ cells per animal was calculated and averaged for each treatment group. For the assessment of descending tract regeneration *in vivo*, the total number of FG^+^ cells within the brainstem on all consecutive brain cross-sections was calculated per animal and averaged for each treatment group. Respective results are given in proportion (fold) to the control group.

For quantitative assessment of posttraumatic astrogliosis (GFAP) as well as inflammation (Iba1) *in vivo*, the immunointensity of the respective marker was quantified on five distinct tissue sections at different distances from the epicenter (0 *μ*m, +/-1200 *μ*m, and +/- 2400 *μ*m from the epicenter) in 6 randomly selected animals of all treatment groups. To this end, images were split into single channels using ImageJ2, ROIs were drawn around the entire spinal cord with exclusion of the cyst, and the “Measure” function was used to output the respective integrated density (immunointensity). Results are given in proportion to the corresponding GFAP- or Iba1-immunointensity in the sham group (fold to sham group). The same 6 animals per group were used to assess the formation of a posttraumatic cyst. To this end, ROIs were drawn around the posttraumatic cystic cavity on 11 GFAP-stained spinal cord sections (0 *μ*m, +/-240 *μ*m, +/-480 *μ*m, +/-720 *μ*m, +/-960 *μ*m, and +/-1200 *μ*m from the epicenter), the area was calculated by applying the “Measure” function, multiplied by the section thickness (30 *μ*m), and then averaged per animal and treatment group. Results are given in mm^3^.

For qualitative assessment of tissue or cell morphology, additional images were taken at 40x magnification (speed of 4).

### 2.8. Statistical Analysis

Results are given as mean ± standard error of the mean (SEM) if not stated otherwise. Normality assumption was evaluated prior to all parametric analyses using Shapiro-Wilk normality tests. For the statistical comparison of means between multiple groups, a unilateral variance analysis (ANOVA) followed by a post hoc Tukey-HSD-test or a Kruskal-Wallis test followed by a post hoc Dunn's test was performed. For the comparison of means between two groups, unpaired two-sample *t*-tests or Mann–Whitney tests were used. A *p* value of *p* < 0.05 was considered significant. All statistical analyses were performed using the software Prism (GraphPad Software, USA) in version 5.

## 3. Results

### 3.1. Proliferation of NPCs *In Vitro*

Proliferation of NPCs was evaluated by quantification of GFP^+^/DAPI^+^ cells after 7 days of incubation with the different growth factor concentrations/combinations (Table 1). The addition of growth factors had increased NPC proliferation in general (Figure 1**(**h)), with groups 2-4 showing higher counts of GFP^+^ cells/mm^2^ compared to the control group (group 1). However, a significant difference in NPC proliferation could only be observed between group 2 and group 1 (155 ± 29 vs. 79 ± 12 GFP^+^/DAPI^+^ cells/mm^2^, *p* = 0.034), while the increased proliferation in group 3 (112 ± 17 GFP^+^/DAPI^+^ cells/mm^2^) and group 4 (150 ± 14 GFP^+^/DAPI^+^ cells/mm^2^) did not reach statistical significance compared to the control group (Figure 1**(**i)). There was no statistically significant difference between the NPC proliferation in the growth factor groups (2-4) either, suggesting that a higher growth factor concentration (group 3) or the addition of a third growth factor (PDGF-AA in group 4) had no additional benefit. We, therefore, concluded, that the growth factor combination and concentration in group 2 (10 ng/ml EGF + 10 ng/ml bFGF) was sufficient to increase NPC proliferation *in vitro*.

### 3.2. Differentiation of NPCs towards the Neuronal Lineage *In Vitro*

**Figure 2:**
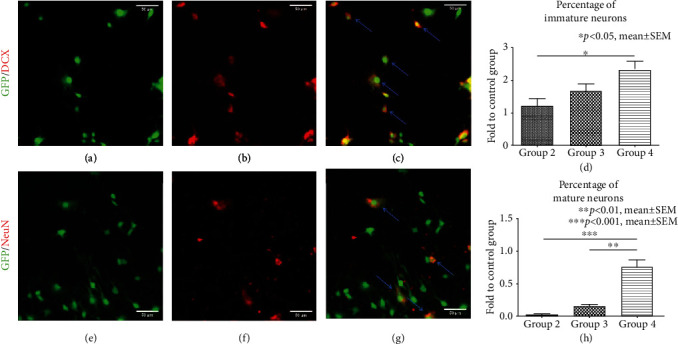
Differentiation of NPCs towards the neuronal lineage *in vitro*. (a-c) Costaining of GFP^+^/DCX^+^ cells (blue arrows) indicating neuronal precursors. (d) Quantification of neuronal precursors after 7 days of incubation with the different growth factor concentrations/combinations in groups 2-4, expressed in proportion to (fold to) the control group (group 1). (e-g) Costaining of GFP^+^/NeuN^+^ cells (blue arrows) indicating mature neurons. (h) Quantification of mature neurons after 7 days of incubation with the different growth factor concentrations/combinations in groups 2-4, expressed in proportion to (fold to) the control group (group 1).

Differentiation of NPCs towards the neuronal lineage was assessed by colocalization of GFP^+^ cells with immunofluorescence markers for neuronal precursors/mature neurons after 7 days of incubation with the different growth factor concentrations/combinations (Table 1). Differentiated neuronal precursors (GFP^+^/DCX^+^ cells, Figures 2(a)–2**(**c)) or mature neurons (GFP^+^/NeuN^+^ cells, Figures 2(e)–2**(**g)) were quantified. Differentiation of NPCs into neuronal precursors (GFP^+^/DCX^+^ cells) was highest in group 4 (2.33 ± 0.25), followed by group 3 (1.67 ± 0.22) and group 2 (1.22 ± 0.22) in proportion to the control group (group 1). A significant difference could be observed with the addition of PDGF-AA to the normal concentration of EGF/bFGF in group 4 compared to the lower concentration of EGF/bFGF in group 2 (*p* = 0.035, Figure 2**(**d)). Similarly, the incubation of NPCs with the three growth factors in group 4 had led to the highest population of mature neurons (GFP^+^/NeuN^+^ cells) in proportion to the control group, followed by group 3 and group 2 (0.76 ± 0.11, 0.15 ± 0.02, and 0.02 ± 0.02, respectively). Moreover, differentiation into mature neurons was significantly increased after incubation with the growth factor combination in group 4 compared to group 3 (*p* = 0.0012) as well as group 2 (*p* < 0.001, Figure 2**(**h)). Overall, differentiation into mature neurons was, however, lower in all growth factor groups compared to the control group. These findings support the conclusion that a growth factor concentration and combination of 20 ng/ml EGF + 20 ng/ml bFGF + 6 ng/ml PDGF − AA (group 4) is most suitable to promote differentiation of NPCs towards the neuronal lineage.

### 3.3. Differentiation of NPCs towards the Oligodendroglial Lineage *In Vitro*

**Figure 3:**
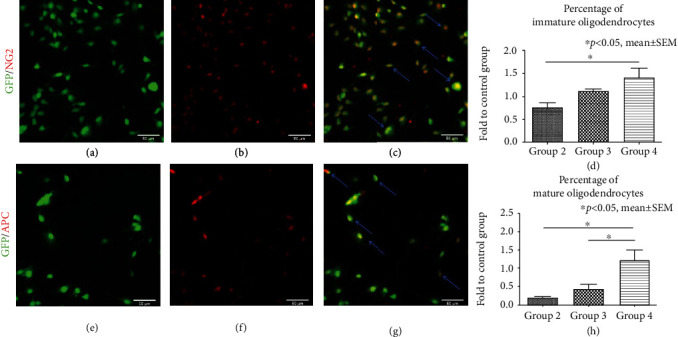
Differentiation of NPCs towards the oligodendroglial lineage *in vitro*. (a-c) Costaining of GFP^+^/NG2^+^ cells (blue arrows) indicating oligodendrocyte precursors. (d) Quantification of oligodendrocyte precursors after 7 days of incubation with the different growth factor concentrations/combinations in groups 2-4, expressed in proportion to (fold to) the control group (group 1). (e-g) Costaining of GFP^+^/APC^+^ cells (blue arrows) indicating mature oligodendrocytes. (h) Quantification of mature oligodendrocytes after 7 days of incubation with the different growth factor concentrations/combinations in groups 2-4, expressed in proportion to (fold to) the control group (group 1).

Colocalization of GFP^+^ cells with the immunofluorescence marker NG2 for oligodendrocyte precursors (Figures 3**(**a)–3**(**c)) and the marker APC for mature oligodendrocytes (Figures 3**(**e)–3**(**g)) was quantified after 7 days of incubation with the different growth factor concentrations/combinations (Table 1) to assess differentiation of NPCs towards the oligodendroglial lineage [12, 32]. Similar to our findings with neuronal precursors, the differentiation of NPCs into oligodendrocyte precursors (GFP^+^/NG2^+^ cells) was highest in group 4 (1.41 ± 0.20), followed by groups 3 and 2 (1.12 ± 0.05 and 0.76 ± 0.10, respectively) in proportion to the control group (group 1).

With the addition of PDGF-AA to the normal concentration of EGF/bFGF in group 4, the number of oligodendrocyte precursors was significantly increased in comparison to the lower concentration of EGF/bFGF in group 2 (*p* = 0.031, Figure 3**(**d)). Moreover, differentiation of NPCs into mature oligodendrocytes (GFP^+^/APC^+^ cells) was also highest in group 4 (1.23 ± 0.28) and thereby not only significantly increased compared to the lower EGF/bFGF concentration in group 2 (0.21 ± 0.03, *p* = 0.015) but also to the normal EGF/bFGF concentration without PDGF-AA in group 3 (0.44 ± 0.12, *p* = 0.045, Figure 3**(**h)) in proprotion to the control group. We, therefore, assume that 6 ng/ml PDGF-AA is enhancing NPC differentiation towards the oligodendroglial lineage and that in combination with 20 ng/ml EGF + 20 ng/ml bFGF (group 4), it results in a high differentiation of NPCs into mature oligodendrocytes *in vitro*.

### 3.4. Differentiation of NPCs into Astrocytes and Undifferentiated NPCs *In Vitro*

**Figure 4:**
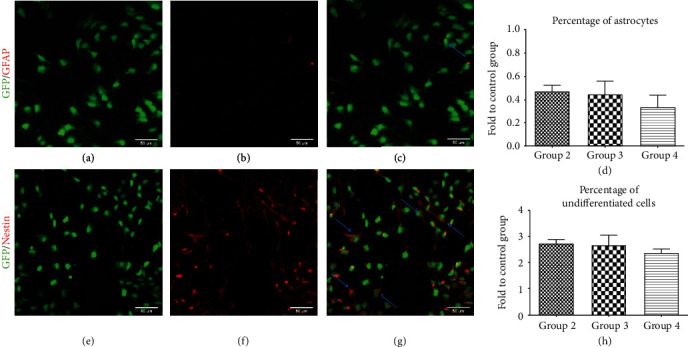
Differentiation of NPCs into astrocytes and quantification of undifferentiated NPCs *in vitro*. (a-c) Costaining of GFP^+^/GFAP^+^ cells (blue arrows) indicating astrocytes. (d) Quantification of astrocytes after 7 days of incubation with the different growth factor concentrations/combinations in groups 2-4, expressed in proportion to (fold to) the control group (group 1). (e-g) Costaining of GFP^+^/Nestin^+^ cells (blue arrows) indicating undifferentiated NPCs. (h) Quantification of undifferentiated NPCs after 7 days of incubation with the different growth factor concentrations/combinations in groups 2-4, expressed in proportion to (fold to) the control group (group 1).

Astrocytic differentiation of NPCs was assessed by quantification of GFP^+^ cells colocalizing with the astrocytic marker GFAP (Figures 4**(**a)–4(c)) after 7 days of incubation with the different growth factor concentrations/combinations (Figures 4**(**a)–4(c)). In proportion to the control group (group 1), the differentiation into astrocytes remained generally low and no significant difference could be observed between the low concentration group (group 2, 0.47 ± 0.06), the normal concentration group (group 3, 0.45 ± 0.10) and even the normal concentration/enhanced combination group (group 4, 0.34 ± 0.10, Figure 4(d)). Similarly, assessment of undifferentiated NPCs, characterized by costaining of GFP with the marker nestin (Figures 4**(**e)-4**(**g)) revealed no significant differences between the different treatment groups either. However, the addition of growth factors had led to a clear increase of undifferentiated NPCs in proportion to the control group in general, with the highest findings in group 2 (2.75 ± 0.13), followed by group 3 (2.69 ± 0.36) and group 4 (2.38 ± 0.15, Figure 4**(**h)). These results suggest that a major portion of NPCs remain undifferentiated when incubated with the different growth factor concentrations and combinations in our study while astrocytic differentiation is generally reduced.

### 3.5. Proliferation and Differentiation of NPCs *In Vivo*

In consideration of our *in vitro* findings with the different growth factor concentrations/combinations, which had revealed the most promising effects on proliferation and differentiation of NPCs for group 4 (20 ng/ml EGF + 20 ng/ml bFGF + 6 ng/ml PDGF-AA), we chose to use this growth factor cocktail to support NPC proliferation and differentiation after SCI *in vivo*. Due to the intrathecal application via an osmotic pump and according to previous reports, the concentration of the growth factors had to be increased by the factor 1,500 for *in vivo* use, resulting in 30 *μ*g/ml EGF, 30 *μ*g/ml bFGF, and 10 *μ*g/ml PDGF-AA [30].

**Figure 5:**
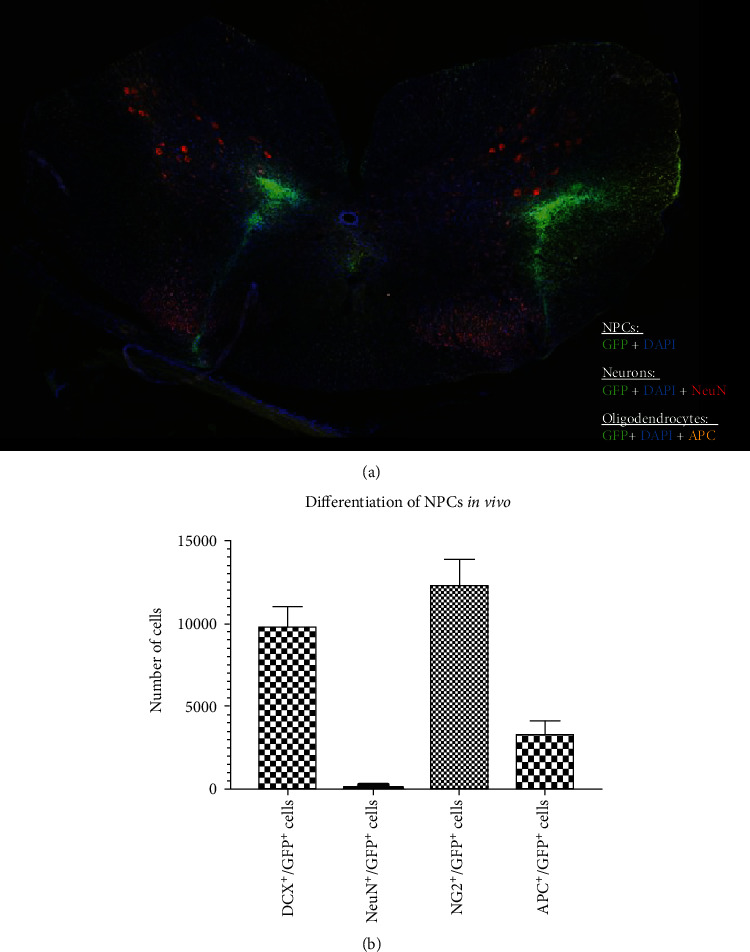
Differentiation of transplanted NPCs *in vivo* 8 weeks after cervical SCI. (a) Spinal cord cross-section showing the spatial distribution of surviving NPCs (GFP^+^/DAPI^+^), NPC-derived neurons (GFP^+^/DAPI^+^/NeuN^+^) and NPC-derived oligodendrocytes (GFP^+^/DAPI^+^/APC^+^). (b) Number of NPC-derived neuronal precursors (DCX^+^/GFP^+^) and mature neurons (NeuN^+^/GFP^+^) as well as oligodendrocyte precursors (NG2^+^/GFP^+^) and mature oligodendrocytes (APC^+^/GFP^+^) in the injured spinal cord of animals in the NPC group (group 1).

At the final stage of the experiment 8 weeks after clip contusion/compression SCI at the C6 level, the survival rate of transplanted NPCs, measured by quantification of GFP^+^/DAPI^+^ cells and expressed as the mean percentage of the initial cell graft given for transplantation was 2.37 ± 0.43% in group 1 animals. NPCs were typically located in the dorsal white or gray matter and a rostrocaudal distribution over a length of 4.63 ± 0.41 mm could be observed (Figure 5(a)). Assessment of the differentiation of surviving NPCs along the neuronal lineage revealed a total number 9,867 ± 1,146 neuronal precursors (GFP^+^/DCX^+^ cells) as well as 223 ± 90 mature neurons (GFP^+^/NeuN^+^ cells). Overall, more NPCs had differentiated towards the oligodendroglial lineage, represented by a total number of 1, 2363 ± 1,515 oligodendrocyte precursors (GFP^+^/NG2^+^ cells) and 3,367 ± 756 mature oligodendrocytes (GFP^+^/APC^+^ cells, Figure 5(b)). These findings suggest that transplanted NPCs are able to survive for several weeks after SCI and predominantly differentiate towards the oligodendroglial lineage with only three growth factors used for intrathecal administration.

### 3.6. Functional Outcome and Descending Tract Regeneration

**Figure 6:**
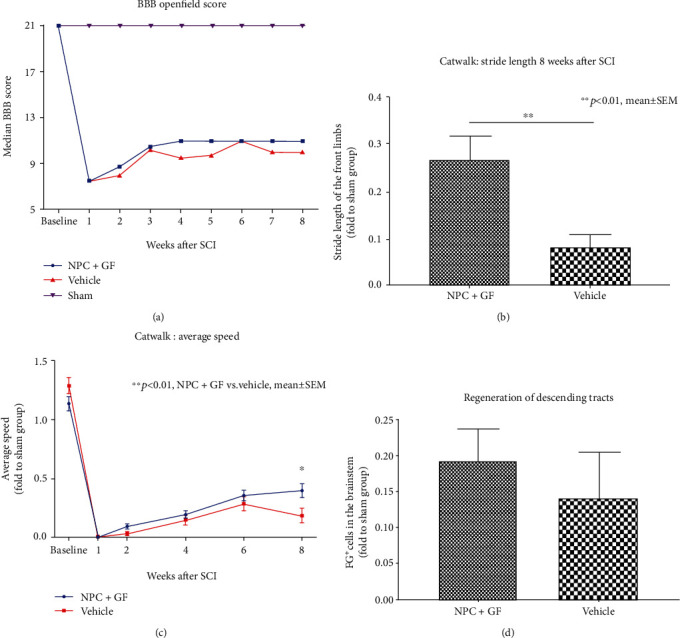
*In vivo* functional recovery and descending tract regeneration after cervical SCI. (a) Weekly median BBB openfield scores. (b) “Stride Length” measurement of the CatWalk gait analysis expressed in proportion to (fold to) the sham group 8 weeks after SCI. (c) “Average Speed” measurement of the CatWalk gait analysis expressed in proportion to (fold to) the sham group over the course of the experiment. (d) FG^+^ neurons as a surrogate marker for descending tract regeneration expressed in proportion to (fold to) the sham group 8 weeks after the injury.

Functional recovery of the hindlimbs was assessed by using the BBB locomotor rating scale (Figure 6(a)). At baseline, all animals showed a BBB score of 21 points. While the BBB score of all sham animals (group 3) remained 21 over the course of the experiment, the C6 contusion/compression SCI proofed to have a devastating impact on the hindlimb function of animals in groups 1 and 2. One week after injury, all BBB scores were lower than 9 points (unable to bear own weight). However, gradual improvement over the course of the experiment could be observed until a median of 11 (6-11) points in the NPC (group 1) and a median of 10 (6-11) points in the vehicle animals (group 2) were reached 8 weeks after SCI. These differences were, however, not significant.

To assess functional recovery of the front limbs, we used the “Stride Length” (SL) measurement of the CatWalk automated quantitative gait analysis system (Figure 6(b)). Because it quantifies the distance between the same paws during a run over the CatWalk runway, increased SL is a sensitive indicator for increased muscle strength of the corresponding limb. At the end of the experiment 8 weeks after SCI, SL values in proportion to the sham group were significantly larger in the NPC animals (group 1) compared to the vehicle animals (group 2; 0.27 ± 0.05 vs. 0.08 ± 0.03, *p* = 0.004). General functional recovery was additionally assessed with the “Average Speed” (AS) measurement in the CatWalk automated quantitative gait analysis system (Figure 6(c)). AS hereby represents the speed with which an animal runs over the CatWalk runway and is a good indicator for the general locomotor function. Starting from week 2 after SCI, animals in the NPC group showed a trend towards faster AS values in proportion to the sham group compared to vehicle animals which remained present over the course of the experiment and reached statistical significance at the last timepoint 8 weeks after SCI (0.39 ± 0.06 vs. 0.18 ± 0.06), *p* = 0.0172).

The extent of descending tract regeneration which is associated with functional recovery can be evaluated by retrograde fiber labeling with Fluorogold (FG). FG is migrating through the axons across the lesion site and can be visualized and quantified in the brainstem 7 days after injection [33]. In proportion to the sham group (group 3), animals in the NPC+GF group (group 1) showed more FG^+^ neurons in their brainstems compared to animals in the vehicle group (group 2; 0.19 ± 0.04 vs. 0.14 ± 0.06), suggesting increased descending tract regeneration. However, this difference did not reach statistical significance (*p* = 0.5151; Figure 6(d)).

### 3.7. Mechanisms of Functional Recovery after NPC Transplantation

To evaluate possible mechanisms of functional recovery after NPC transplantation, several secondary injury changes 8 weeks after SCI were assessed.

**Figure 7:**
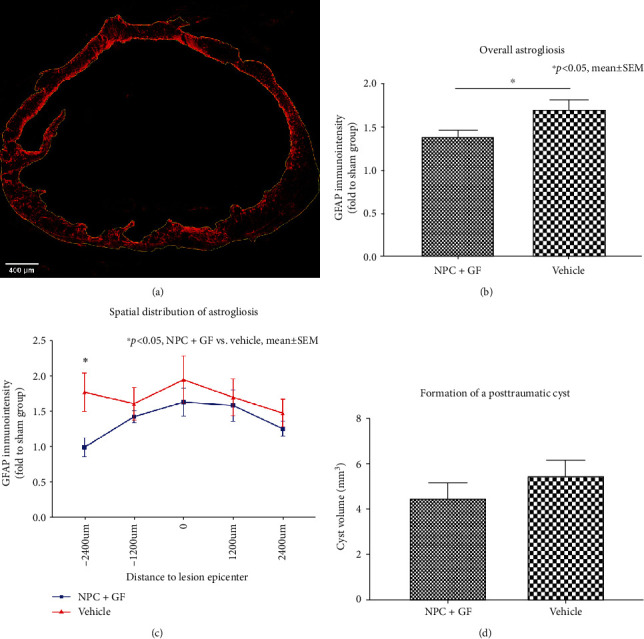
*In vivo* astrogliosis and cyst formation 8 weeks after cervical SCI. (a) Spinal cord cross-section stained for GFAP (red) with ROIs (yellow) drawn around the entire spinal cord as well as the intramedullary, posttraumatic cyst (10x magnification). (b) Overall GFAP-immunointensity and (c) spatial distribution of GFAP-immunointensity expressed in proportion to (fold to) the sham group as a marker for reactive astrogliosis in the injured spinal cord. (d) Volume of the posttraumatic intramedullary cyst.

Reactive astrogliosis prevents the regeneration of axons and is a key component of the secondary injury cascade after SCI. Interestingly, in proportion to the control group (group 3), overall astrogliosis measured as the immunointensity of GFAP (Figure 7(a)) was significantly lower in animals that had received NPC+GF (group 1; 1.385 ± 0.08) compared to vehicle animals (group 2; 1.71 ± 0.11; *p* = 0.022; Figure 7(b)). Spatial distribution of astrogliosis revealed the biggest difference between NPC and vehicle animals at the most caudal distance to the lesion epicenter (-2,400 *μ*m; 1.00 ± 0.13 vs. 1.78 ± 0.27, *p* = 0.0284, Figure 7(c)). The formation of a cystic cavity in the injured spinal cord represents another important pathophysiological process after SCI and acts as a physical barrier for axonal regeneration and sprouting. Such cystic cavities were present in all injured animals after SCI (Figure 7(a)) and were generally smaller in the NPC + GF group (group 1) compared to the vehicle group (group 2; 4.47 ± 0.69 mm^3^ vs. 5.49 ± 0.67 mm^3^) without reaching a statistically significant difference (*p* = 0.2222; Figure 7(d)).

**Figure 8:**
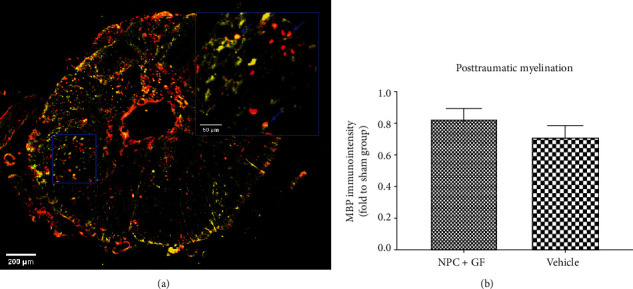
*In vivo* posttraumatic myelination 8 weeks after cervical SCI. (a) Spinal cord cross-section stained for APC (red), a marker for mature oligodendrocytes and MBP (yellow), depicting myelination (10x magnification). Inlay (blue) with arrows (blue) showing the spatial relationship of MBP and APC in the injured spinal cord (40x magnification). (b) Overall MBP-immunointensity as a surrogate marker for posttraumatic myelination expressed in proportion to (fold to) the sham group.

Chronic progressive demyelination contributes to the functional deficits occurring after SCI [34]. Quantification of the immunointensity of MBP, which is expressed by mature oligodendrocytes to maintain the correct structure of myelin (Figure 8(a)), revealed an increased extent of posttraumatic myelination in the injured spinal cord after treatment with NPC+GF (group 1) compared to untreated animals (group 2; 0.83 ± 0.07 vs. 0.71 ± 0.07 in proportion to the sham group) without reaching a statistically significant difference (*p* = 0.2818; Figure 8(b)).

**Figure 9:**
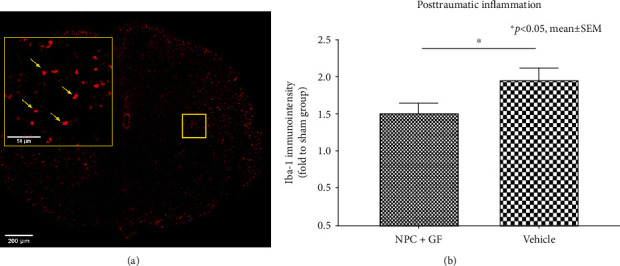
*In vivo* posttraumatic inflammation 8 weeks after cervical SCI. (a) Spinal cord cross-section stained for Iba1 (red), a marker for microglia/macrophages (10x magnification). Inlay (yellow) with arrows (yellow) depicting the cell morphology (40x magnification). (b) Quantification of Iba-1-immunointensity as a surrogate marker for posttraumatic inflammation, expressed in proportion to (fold to) the sham group.

**Figure 10:**
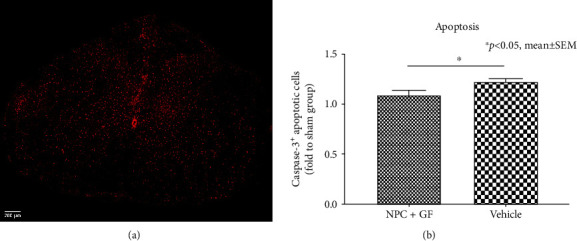
*In vivo* apoptotic cell death 8 weeks after cervical SCI. (a) Spinal cord cross-section stained for Caspase-3 (red), a marker for ongoing apoptosis. (b) Quantification of Caspase-3^+^, apoptotic cells in the injured spinal cord in proportion to (fold to) the sham group.

The inflammatory response induced by SCI plays an important role in the prevention of neuroregeneration and can persist till chronic stages. Quantification of the immunointensity of Iba1^+^ macrophages in the injured spinal cord (Figure 9(a)) revealed a significantly reduced extent of posttraumatic inflammation in the NPC+GF group (group 1) compared to the vehicle group (group 2; 1.52 ± 0.12 vs. 1.96 ± 0.15 in proportion to the sham group; *p* = 0.0149; Figure 9(b)). Accordingly, animals in the NPC+GF group (group 1) showed significantly less Caspase-3^+^ apoptotic cells in the chronic stage after SCI (Figure 10(a)) compared to vehicle animals (group 2; 1.09 ± 0.46 vs. 1.22 ± 0.04 in proportion to the sham animals; *p* = 0.0410; Figure 10(b)).

## 4. Discussion

The survival and differentiation of exogenous NPCs after transplantation into the injured spinal cord in animal models is reported to be challenging [1]. To improve the fate of the cell transplant and ultimately enhance functional recovery, researchers have used various experimental settings and modified different aspects of the stem cell treatment including the amount of the transplanted cells or the timepoint of transplantation [2]. However, many studies involving NPC grafting *in vivo* rely on the additional application of growth factors, which are considered to be a mandatory cotreatment by some groups [8].

In their well-recognized experiments, Lu et al. for example successfully used a cocktail of 10 different growth factors to support survival of transplanted NPCs after T3 complete transection [12, 35] and C5 lateral hemisection [13] SCI in rats. Given the high concentrations, combined with the costs of the growth factors applied in such *in vivo* studies, it becomes clear that the financial burden for researchers is huge and might become even greater when translation of findings into the clinical practice is considered. Interestingly, Lu et al. addressed this issue in a recent publication themselves, stating that a reduced number of growth factors would be more practical for clinical translation of their findings. Using a C5 lateral hemisection SCI model, they were able to reduce their grafting “cocktail” to 4 growth factors (BDNF, bFGF, vascular endothelial growth factor, and the cell death inhibitor MDL28170), still able to support survival of grafted NPCs as well as differentiation, thereby even enhancing certain features of growth factor application in general such as a more homogenous cell distribution [14].

In our current study, we firstly assessed the use of an even reduced growth factor cocktail, consisting of either EGF and bFGF in a lower (group 2) and higher (group 3) concentration or a combination of EGF, bFGF, and PDGF-AA (group 4) *in vitro*. We could show that the differentiation of NPCs towards the neuronal and even more the oligodendroglial lineage was most clearly increased with the combination of three growth factors at a higher concentration in group 4, which in addition also enhanced NPC survival and decreased astrocytic differentiation.

Consequently, we translated these findings into an *in vivo* model of rodent cervical clip contusion/compression SCI at the C6 level, which is associated with severe morbidity and mortality of the injured animals, thus reflecting the clinical reality of human SCI [11, 36]. Moreover, inflammation, astrogliosis, or the formation of a proteoglycan scar in the subacute as well as the formation of a cystic cavity in the chronic phase are common features of this injury model, contributing to the chemical as well as physical barriers that prevent endogenous and exogenous neuroregeneration [37]. Despite this hostile microenvironment, our treatment approach with NPC grafting in the subacute phase 10 days after SCI, immunosuppressive medication, and above all continuous, intrathecal administration of the three growth factors EGF, bFGF, and PDGF-AA for one week led to verifiable survival of the transplanted NPCs even in the chronic phase 8 weeks after injury. Moreover, with our “cocktail” of only three growth factors, we could predominantly observe differentiation of NPCs towards the oligodendroglial lineage *in vitro* as well as *in vivo*. This finding is of particular importance, because loss of oligodendrocytes following SCI usually results in demyelination and axonal dysfunction [38] while oligodendroglial cell replacement has been associated with, e.g., enhanced axonal ensheathment and remyelination of the axons in the corticospinal tract, contributing to improved functional recovery [39, 40]. In correspondence to previous reports [41, 42], oligodendroglial differentiation is thereby most likely mediated by PDGF-AA, which is known to promote the proliferation of oligodendrocyte progenitor cells [43] as can been seen in our own *in vitro* results. Surprisingly, the regeneration of descending tracts and the extent of posttraumatic myelination were not significantly increased 8 weeks after SCI in our *in vivo* experiment despite the presence of NPC-derived oligodendrocytes, suggesting that the surviving NPC-graft might be too small.

Similar to our findings, several authors have reported low differentiation of transplanted NPCs into neurons in animal models of severe SCI *in vivo*, even when growth factor administration was combined with additional treatment strategies such as self-assembling peptides [11]. This reduced neuronal differentiation has been linked to an increased astrocytic differentiation of transplanted NPCs, mediated by the microenvironment of the injured spinal cord [44, 45]. Correspondingly, studies on the fate of endogenous NPCs have shown increased astrocytic differentiation after SCI, contributing to glial scar formation and astrogliosis [46]. In our study, however, the growth factors EGF, bFGF, and PDGF-AA were able to reduce astrocytic differentiation of NPCs *in vitro*. Moreover, astrogliosis around the injury site was decreased after transplantation of NPCs and administration of the growth factors *in vivo*, possibly limiting its well-established inhibitory influence on axonal regeneration and plasticity after SCI [47, 48]. Because we could already observe a low differentiation of NPCs towards the neuronal lineage *in vitro*, with even a considerable decrease of mature neurons for the different growth factor concentrations/combinations compared to the control group, a direct effect of the growth factors selected in our study on the differentiation pattern of NPCs seems more plausible. This confirms reports of other authors, who found a similar differentiation pattern of transplanted NPCs with the use of the growth factors EGF, bFGF and PDGF-AA after SCI *in vivo* [8].

Although some researchers have claimed that functional recovery seen after SCI in animal models is dependent on the neuronal differentiation of transplanted NPCs [49, 50] which allow the formation of synapses with the injured axonal tracts or host interneurons [51], transplantation strategies relying primarily on oligodendroglial differentiation have shown to improve neurologic outcome as well [39, 52]. Correspondingly, increased oligodendroglial differentiation of transplanted NPCs and possibly the indirect effects of undifferentiated NPCs and monopotential precursors led to partial improvement of functional recovery 8 weeks after severe cervical SCI in our study as well. Although the exact mechanisms of NPC-mediated functional recovery are not yet fully resolved, our findings suggest that the decrease of apoptotic cell death which is normally a common feature of traumatic insults to the CNS [53] as well as the reduced amount of macrophages which usually are a decisive immune cell population in chronic inflammatory stages after SCI [54] might play an important role.

With regards to the efforts associated with NPC transplantation and administration of the growth factors, the effects observed on locomotor regeneration are, however, still low. For the grafted cells to adopt both, the oligodendroglial as well as the neuronal cell fate, our transplantation strategy with only three growth factors could be improved by synergistic treatments such as physical therapy which has shown to promote neuronal differentiation and facilitate synaptic connectivity [55] in future experiments.

## 5. Conclusions

In our current study, we identified a cocktail of three growth factors (EGF, bFGF and PDGF-AA) suitable to increase proliferation of NPCs and direct differentiation towards the oligodendroglial and the neuronal lineage while reducing astrocytic differentiation *in vitro*. When using this comparatively small number of growth factors for intrathecal administration together with NPC transplantation after cervical SCI *in vivo*, survival of NPCs could be observed 8 weeks after injury with a majority of the transplanted cells differentiating into oligodendrocytes or oligodendrocytic precursors. In addition, functional recovery was partially increased with NPC transplantation and administration of the growth factors, possibly related to decreased posttraumatic inflammation and apoptotic cell death in the injured spinal cord. In comparison to reports of extensive growth factor use in the literature, this reduced three-growth factor cocktail seems to be sufficient to support NPC-transplantation into the hostile postinjury environment. However, synergistic treatment approaches helping to promote neuronal differentiation might be necessary to further improve functional recovery after cervical SCI.

## Figures and Tables

**Table 1:** Different concentrations/combinations of growth factors used *in vitro*.

Group no.	1^st^ growth factor (concentration)	2^nd^ growth factor (concentration)	3^rd^ growth factor (concentration)
1	—	—	—
2	EGF (10 ng/ml)	bFGF (10 ng/ml)	—
3	EGF (20 ng/ml)	bFGF (20 ng/ml)	—
4	EGF (20 ng/ml)	bFGF (20 ng/ml)	PDGF-AA (6 ng/ml)

EGF = epidermal growth factor; bFGF = basic fibroblast growth factor; PDGF-AA = platelet-derived growth factor AA.

## Data Availability

All data used to support the findings of this study are available from the corresponding author upon reasonable request.
